# Green-synthesized zinc oxide nanoparticles from *Garcinia mangostana* leaf extract: A promising antibacterial agent for poultry

**DOI:** 10.14202/vetworld.2025.263-269

**Published:** 2025-02-08

**Authors:** R. K. Rusli, Zurmiati Zurmiati, R. Mutia, S. Reski, A. Darmawan, C. Hidayat, M. E. Mahata, M. Hilmi, A. Yuniza

**Affiliations:** 1 Department of Nutrition and Feed Technology, Faculty of Animal Science, Universitas Andalas, Padang, 25175, Indonesia; 2 Department of Nutrition and Feed Technology, Faculty of Animal Science, IPB University, Bogor, 16680, Indonesia; 3 Research Center for Animal Husbandry, The National Research and Innovation Agency, Jakarta, 10340, Indonesia; 4 Study Program of Livestock Product Processing Technology, Politeknik Negeri Banyuwangi, Banyuwangi, 68461, Indonesia

**Keywords:** antibacterial, green synthesis, nanotechnology, poultry health, zinc oxide nanoparticles

## Abstract

**Background and Aim:**

The development of zinc-based antimicrobials has progressed from conventional to nanoparticle (NP) formulations due to their enhanced biological properties. This study evaluates the antibacterial efficacy of green-synthesized zinc oxide NPs (ZnO-NPs) derived from *Garcinia mangostana* leaf extract against both pathogenic and non-pathogenic bacteria relevant to poultry health.

**Materials and Methods:**

Using a completely randomized design, six treatment groups were tested: Positive control (PC) (tetracycline, 2.5 mg/mL), negative control (NC) (HCl 0.1 N + distilled water), and ZnO-NPs at concentrations of 2.5, 5, 7.5, and 10 mg/mL. The antimicrobial activity was assessed using the agar well diffusion method, measuring inhibition zones against *Escherichia coli, S*. Typhimurium, *Staphylococcus aureus*, and *Lactobacillus plantarum.*

**Results:**

The positive control demonstrated the most significant inhibition zones across all bacterial strains. ZnO-NPs exhibited dose-dependent antibacterial activity, with maximum inhibition zones recorded as 18.58 mm for E. *coli*, 17.09 mm for S. Typhimurium, and 17.41 mm for *S. aureus* at the highest concentration (10 mg/mL). However, the antibacterial activity against *L. plantarum* was less pronounced, with a maximum inhibition zone of 9.93 mm. These findings indicate selective toxicity of ZnO-NPs, favoring pathogenic over non-pathogenic strains.

**Conclusion:**

Green-synthesized ZnO-NPs using *G. mangostana* leaf extract demonstrate promising antibacterial properties against poultry pathogens. Their selective action highlights potential applications as eco-friendly feed additives to improve poultry health and mitigate pathogenic risks.

## INTRODUCTION

Bacteria play an important role in maintaining digestive health and the overall well-being of poultry. *Escherichia coli* and *Salmonella* spp. are pathogenic bacteria that can be found in the intestines of animals [1]. In poultry, *E. coli* is the main causative agent of colibacillosis [2], whereas *Salmonella* spp. is associated with enteritis and systemic infections [3], both of which can cause significant economic losses in the poultry industry. With advancements in materials technology, nanotechnology – especially nanoparticles (NPs) – has developed rapidly. NPs play an important role in veterinary science by improving disease diagnosis, treatment, and prevention, thereby improving animal health and welfare [4]. Diverse nanomaterials, including gold and iron oxide NPs, have been utilized to create diagnostic assays capable of swiftly detecting diseases such as avian influenza and Newcastle disease [5, 6], and silver and copper NPs are effective against pathogens such as *Staphylococcus aureus* and *E. coli* [7].

In recent years, zinc research and development have shifted from conventional forms to NP forms, one of which involves green synthesis methods that utilize plant components such as roots, stems, fruits, leaves, and seeds [8]. Zinc oxide (ZnO) has attracted significant interest from researchers in the field of metal NPs. ZnO-NPs stand out for their outstanding characteristics, including their antibacterial [9, 10], anticoccidial, antioxidant, and anti-inflammatory [11], and ability to reduce heat stress in poultry [12]. Various plant extracts have been used to prepare ZnO-NPs, including *Garcinia mangostana* [13, 14], green algae [15], *Origanum majorana* [16], *Myristica fragrans* [17], *Zingiber officinale*, and *Allium sativum* [18].

Mangosteen leaf extract (*G. mangostana*) is a potential plant extract for use in the manufacture of zinc NPs; this is because mangosteen leaf extract contains phytochemical compounds such as flavonoids [19], tannins, total phenols [19, 20], and saponins [20]. These compounds are essential as capping, stabilizing, and reducing agents during the manufacturing of environmentally friendly NPs [14, 21]. However, information on the use of ZnO-NPs synthesized from mangosteen leaf extract as antibacterial agents in poultry still needs to be available.

Therefore, this study aimed to evaluate the effectiveness of ZnO-NPs against the most common pathogenic (*E. coli*, *Salmonella* Typhimurium, and *S. aureus*) and non-pathogenic (*Lactobacillus plantarum*) bacteria in the digestive tract of poultry through *in vitro* studies.

## MATERIALS AND METHODS

### Ethical approval

Ethics approval was not required for this study as all experiments were performed *in vitro*.

### Study period and location

The study was conducted from June 2024 to October 2024 at the Nonruminant Nutrition Laboratory, Faculty of Animal Science, Universitas Andalas, Indonesia.

### Aqueous extracts of *G. mangostana* leaves

*G. mangostana* leaves were obtained from a plantation in Koto Lua village, Padang City, West Sumatra, Indonesia and extracted using the procedure described by Rusli *et al*. [19]. *G. mangostana* leaves (shoots) were washed thoroughly with running water to remove dust and other impurities. The leaves of *G. mangostana* were dried for 24 h in an oven at 50°C for 24 h. After drying, *G. mangostana* leaves were ground and filtered (355 µm, Endecotts, Ltd., London, England). *G. mangostana* leaf flour (10 g) was mixed with distilled water (100 mL) and then heated at 50°C for 45 min. After cooling, *G. mangostana* leaf extract was filtered twice using Whatman paper No. 1 (Cytiva, China). The mangosteen leaf extraction was stored at 4°C.

### Green synthesis of NPs

The chemical compound zinc nitrate hexahydrate (Zn(NO_3_)_2_.6H_2_O) was used in this study to synthesize NPs using the method of Rusli *et al*. [14]. In total, 50 mL of aqueous extract of *G. mangostana* leaves was put into a glass beaker, heated at 70°C, and stirred. Then, 4 g of zinc nitrate hexahydrate was slowly added. As the reaction progressed, the color of the solution gradually changed until a reddish-brown paste was formed. The paste was then transferred to a ceramic crucible and heated in a furnace at 300°C for 2 h. The obtained off-white powder was used in further research. Subsequently, a size particle analyzer was employed to measure the particle size, where the particle size distribution value in this study was 641.97 ± 270.35 nm.

### Preparation and antibacterial assay

Antibacterial preparations and tests were based on Kusumaningrum *et al*. [22]. *E. coli*, *S*. Typhimurium, and *S. aureus* were each inoculated 0.1 mL into 100 mL of Mueller–Hinton agar (MHA) media (Oxoid, Ireland). In comparison, *L. plantarum* was inoculated 0.1 mL into 100 mL of Man–Rogosa–Sharpe Agar (MRSA) media (Oxoid) and then incubated overnight. Next, 15–20 mL of MHA and MRSA media containing the test cultures were poured into sterile Petri dishes and allowed to solidify. After solidification, the antibacterial activity was tested using the agar well diffusion method. The media were punched into as many as six holes (wells) with diameters of about 6 mm. Each hole was inoculated with 60 µL of the treatment solution (positive control [PC], negative control [NC], Z1, Z2, Z3, and Z4). Then, the cells were incubated at 37°C for 24 h, and the diameter of the inhibition zone was formed. This study used solvents to dissolve ZnO-NPS, namely 0.1 N HCl + Aquadest. The inhibition zone is the precise area formed around the wells, as measured by a millimeter-scale caliper [23].

### Experimental design

This research used a completely randomized design with six treatments: (PC, tetracycline 2.5 mg/mL^-1^); NC (HCl 0,1 N + Aquadest); Z1 (ZnO-NPs 2.5 mg/mL^-1^); Z2 (ZnO-NPs 5 mg/mL^-1^); Z3 (ZnO-NPs 7.5 mg/mL^-1^); and Z4 (ZnO-NPs 10 mg/mL^-1^). Each treatment was repeated 3 times.

### Statistical analysis

The data collected, representing inhibition zones (in mm), were analyzed using a one-way analysis of variance to assess the effect of treatments on bacterial growth inhibition. Differences between treatment means were further evaluated using Duncan’s multiple range test, ensuring rigorous post-hoc comparisons. All statistical tests were conducted at a significance level of p < 0.01, and results are expressed as means ± standard error of the mean.

## RESULTS

**Figure 1:**
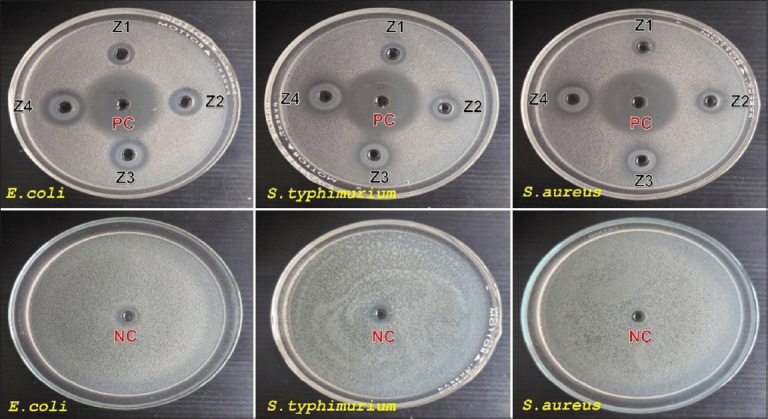
Zone of inhibition of ZnO-NPs by Aqueous Extract of *Garcinia mangostana* against bacteria *Escherichia coli*, *Salmonella* Typhimurium, and *Staphylococcus aureus*). PC=Positive control, tetracycline 2.5 mg/mL^-1^, NC=Negative control, HCl 0,1 N + Aquadest; Z1 (ZnO-NPs 2.5 mg/mL^-1^), Z2 (ZnO-NPs 5 mg/mL^-1^), Z3 (ZnO-NPs 7.5 mg/mL^-1^), and Z4 (ZnO-NPs 10 mg/mL^-1^), ZnO-NPs=Zinc oxide nanoparticles.

**Figure 2:**
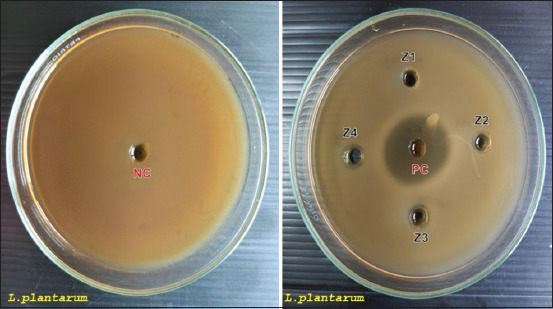
Zone of inhibition of ZnO-NPs by aqueous extract of *Garcinia mangostana* against bacteria (*Lactobacillus plantarum*). PC=Positive control, tetracycline 2.5 mg/mL^-1^, NC=Negative control, HCl 0,1 N + Aquadest, Z1 (ZnO-NPs 2.5 mg/mL^-1^), Z2 (ZnO-NPs 5 mg/mL^-1^), Z3 (ZnO-NPs 7.5 mg/mL^-1^), and Z4 (ZnO-NPs 10 mg/mL^-1^), ZnO-NPs=Zinc oxide nanoparticles.

Figure 1 shows the inhibition zones generated against pathogenic bacteria, and Figure 2 shows the inhibition zones against non-pathogenic bacteria. The inhibition zone diameters (mm) for various strains of pathogenic bacteria are presented in Tables 1-3, while Table 4 presents the inhibition zone diameters (mm) for non-pathogenic bacteria.

**Table 1:** Zone of inhibition of ZnO-NPs by aqueous extract of *Garcinia mangostana* against *Escherichia coli*.

Treatment	Zone of inhibition (mm)
PC	31.57^a^
NC	0.00^f^
Z1	11.45^e^
Z2	13.94^d^
Z3	16.87^c^
Z4	18.58^b^
Standard error of the mean	2.28
p-value	< 0.001

PC=Positive control, tetracycline 2.5 mg/mL^-1^, NC=Negative control, HCl 0.1 N+Aquadest, Z1 (ZnO-NPs 2.5 mg/mL^-1^), Z2 (ZnO-NPs 5 mg/mL^-1^), Z3 (ZnO-NPs 7.5 mg/mL^-1^), and Z4 (ZnO-NPs 10 mg/mL^-1^), ZnO-NPs=Zinc oxide nanoparticles. ^a-f^ Means with different superscripts in the same column differ significantly (p < 0.01)

**Table 2:** Zone of inhibition of ZnO-NPs by aquoes extract of *Garcinia mangostana* against *Salmonella* Typhimurium.

Treatment	Zone of inhibition (mm)
PC	31.59^a^
NC	0.00^f^
Z1	11.31^e^
Z2	13.15^d^
Z3	14.71^c^
Z4	17.09^b^
Standard error of the mean	2.26
p-value	< 0.001

PC=Positive control, tetracycline 2.5 mg/mL^-1^, NC=Negative control, HCl 0.1 N + Aquadest, Z1 (ZnO-NPs 2.5 mg/mL^-1^), Z2 (ZnO-NPs 5 mg/mL^-1^), Z3 (ZnO-NPs 7.5 mg/mL^-1^), and Z4 (ZnO-NPs 10 mg/mL^-1^), ZnO-NPs=Zinc oxide nanoparticles. ^a-f^ Means with different superscripts in the same column differ significantly (p < 0.01)

**Table 3:** Zone of inhibition of ZnO-NPs by aqueous extract of *Garcinia mangostana* against *Staphylococcus aureus*.

Treatment	Zone of inhibition (mm)
PC	32.14^a^
NC	0.00^f^
Z1	10.63^e^
Z2	13.19^d^
Z3	15.01^c^
Z4	17.41^b^
Standard error of the mean	2.32
P-value	< 0.001

PC=Positive control, tetracycline 2.5 mg/mL^-1^, NC=Negative control, HCl 0.1 N + Aquadest, Z1 (ZnO-NPs 2.5 mg/mL^-1^), Z2 (ZnO-NPs 5 mg/mL^-1^), Z3 (ZnO-NPs 7.5 mg/mL^-1^), and Z4 (ZnO-NPs 10 mg/mL^-1^), ZnO-NPs=Zinc oxide nanoparticles. ^a-f^ Means with different superscripts in the same column differ significantly (p < 0.01)

**Table 4:** Zone of inhibition of ZnO-NPs by aqueous extract of *Garcinia mangostana* against *Lactobacillus plantarum*.

Treatment	Zone of inhibition (mm)
PC	24.27^a^
NC	0.00^e^
Z1	0.00^e^
Z2	7.27^d^
Z3	8.54^c^
Z4	9.93^b^
Standard error of the mean	1.97
p-value	< 0.001

PC=Positive control, tetracycline 2.5 mg/mL^-1^, NC=Negative control, HCl 0.1 N + Aquadest, Z1 (ZnO-NPs 2.5 mg/mL^-1^), Z2 (ZnO-NPs 5 mg/mL^-1^), Z3 (ZnO-NPs 7.5 mg/mL^-1^), and Z4 (ZnO-NPs 10 mg/mL^-1^), ZnO-NPs=Zinc oxide nanoparticles. ^a-f^ Means with different superscripts in the same column differ significantly (p < 0.01)

The results revealed that the PC showed inhibition zone activity on all bacterial strains, whereas the NC showed no inhibition zone. ZnO-NPs showed significant antibacterial activity against pathogenic bacteria, with maximum inhibition zones of 18.58 mm for *E. coli*, 17.09 mm for *S*. Typhimurium, and 17.41 mm for *S. aureus*. In addition, this study also found that ZnO-NPs had lower antibacterial activity against nonpathogenic bacteria (*L. plantarum*), with a maximum inhibition zone of only 9.93 mm.

## DISCUSSION

This study identified a significant positive correlation between increasing ZnO-NPs concentrations and increasing inhibition zone diameters for various bacterial strains. These experimental results also show that ZnO-NPs have more potent antibacterial activity against Gram-negative bacteria, including *E. coli* and *S*. Typhimurium, than Gram-positive bacteria, such as *S. aureus* and *L. plantarum*. These results are close to previously reported findings by Rambabu *et al*. [24], and Aldeen *et al*. [25], who showed that Gram-negative bacteria are more sensitive to ZnO-NPs than Gram-positive bacteria. In contrast, Belay *et al*. [26] and Mwafy *et al*. [27] reported that ZnO-NPs exhibit greater antibacterial activity against Gram-positive than Gram-negative bacteria. This difference in sensitivity is consistent with existing literature, which attributes it to structural variations in the cell walls of the two groups of bacteria. Gram-positive bacteria have thicker cell walls rich in peptidoglycan, which is a physical barrier to the penetration of antibacterial agents, making them more resistant than Gram-negative bacteria [24]. Furthermore, the cytotoxicity of nanostructures is greatly influenced by their shape, size, and surface charge, where sharp nanostructures can strongly damage cell membranes and cause cell death [28].

**Figure 3:**
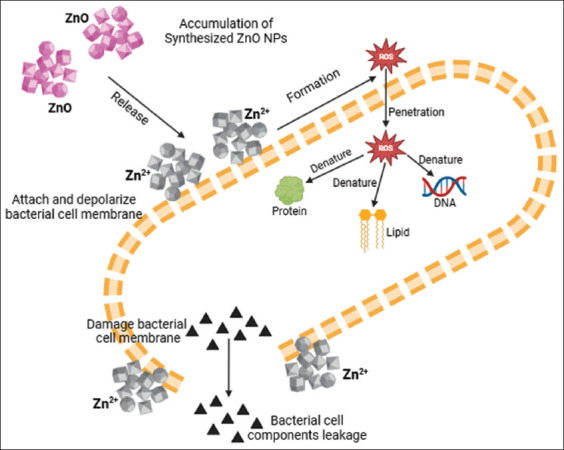
Antibacterial mechanism of zinc oxide nanoparticles (Modified from Chan *et al*. [21]).

Furthermore, the mechanism of the antibacterial activity of ZnO-NPs can be explained through several different molecular pathways (Figure 3) [21]. Studies by Saeed *et al*. [10], Aldeen *et al*. [25], and Chan *et al*. [21] suggested that these mechanisms include the following: (1) Accumulation of ZnO-NPs on the bacterial cell surface, leading to structural damage to the cell membrane and release of intracellular components, which is indicative of membrane dysfunction; (2) formation of Zn^2²^ ions and electrostatic interactions with cell wall components, which increase membrane permeability and allow deeper penetration of NPs into cells, further disrupting important cellular components; and (3) production of reactive oxygen species, which are capable of inducing oxidative stress by damaging DNA, proteins, and membrane lipids, thus triggering apoptosis or lysis of bacterial cells.

## CONCLUSION

This study evaluated the antibacterial activity of green-synthesized ZnO-NPs using *G. mangostana* leaf extract against pathogenic and non-pathogenic bacteria relevant to poultry health. The results demonstrated that ZnO-NPs exhibited significant dose-dependent antibacterial activity against pathogenic bacteria, including *E. coli* (18.58 mm inhibition zone), *S*. Typhimurium (17.09 mm), and *S. aureus* (17.41 mm) at the highest concentration (10 mg/mL). In contrast, the activity against the non-pathogenic *L. plantarum* was comparatively lower (9.93 mm). These findings suggest the selective toxicity of ZnO-NPs toward pathogenic strains, underscoring their potential as eco-friendly antimicrobial agents for poultry applications.

The study’s strengths include its eco-friendly synthesis approach using *G. mangostana* leaf extract, which is cost-effective, sustainable, and reduces environmental harm. ZnO-NPs demonstrated effective antibacterial activity against multiple pathogenic strains, highlighting their potential as feed additives for poultry, aiming to improve gut health and mitigate economic losses caused by bacterial infections.

However, the study has limitations, as it was conducted *in vitro* and may not fully replicate the complex environment of a poultry digestive system. In addition, only a limited spectrum of bacteria was tested, and further characterization of ZnO-NPs regarding their stability, bioavailability, and long-term effects is necessary.

Future studies should focus on evaluating the efficacy and safety of ZnO-NPs in live poultry to validate their practical applications. Investigating the molecular pathways of ZnO-NPs’ antibacterial activity could provide deeper insights and optimize their formulation. Expanding the evaluation to include other pathogenic and non-pathogenic strains could validate the broader utility of ZnO-NPs in veterinary medicine. Moreover, assessing the environmental impact of ZnO-NPs post-use in poultry farming would ensure their safe integration into industry practices.

This research establishes a foundation for the development of ZnO-NPs as effective antibacterial agents in poultry, emphasizing their potential to reduce reliance on conventional antibiotics while promoting sustainable livestock management.

## AUTHORS’ CONTRIBUTIONS

RKR, CH, ZZ, and MH: Conceptualized, methodology and drafted the manuscript. RKR, ZZ, SR, and AD: Analysed and interpreted data. RM, MEM, and AY: Supervised the study and critically revised the manuscript for important intellectual content. All authors have read and approved the final manuscript
